# Challenges for staff encountering older people's existential concerns: Swedish first‐line managers' views. A cross‐sectional study

**DOI:** 10.1111/scs.13300

**Published:** 2024-11-20

**Authors:** Malin Sundström, Kerstin Blomqvist, Margareta Rämgård, Anna‐Karin Edberg

**Affiliations:** ^1^ The Research Platform for Collaboration for Health, Faculty of Health Science Kristianstad University Kristianstad Sweden; ^2^ Faculty of Health and Society Malmö University Malmö Sweden

**Keywords:** existential concerns, first‐line managers, older people, staff, support

## Abstract

**Aims and Objectives:**

To describe first‐line managers' (FLMs) views of the challenges faced by staff when encountering older people's existential concerns in home and residential care.

**Methodological Design and Justification:**

This cross‐sectional study uses a combination of qualitative and quantitative methods.

**Ethical Issues and Approval:**

The study was approved by the Swedish Ethical Review Authority (ref. number 2014/652 ) and followed the guidelines of the Helsinki Declaration.

**Research Methods:**

FLMs in home and residential care were randomly selected and invited to participate. A self‐administered questionnaire was distributed online to 467 managers, 136 (29%) of whom completed it.

**Results:**

About 80% of the FLMs reported that their staff members sometimes or frequently had conversations with the older people in their care about existential concerns, including the following topics: thoughts and feelings about meaning in life; losses and longing for meaningful relations; death, fears and uncertainty and supporting meaningful everyday life. About 75% of the managers also reported that their staff sometimes or often had conversations about existential concerns with one another. Major hindrances to existential conversations were reportedly cognitive impairment and aphasia among the older people and insecurity and unwillingness among the staff. Most managers (73%) reported that their staff received support when encountering existential concerns, mostly as individual support from managers or registered nurses.

**Study Limitations:**

The main limitation of this study is its low response rate, which is common for digital surveys. Nevertheless, the sample is considered to be representative; therefore, the study holds exploratory value.

**Conclusion:**

Regular conversations among staff, improved conversational skills and the ability to listen reflect on and perceive older people's perspective and life world are needed when encountering older people's existential concerns. FLMs play a crucial role in emphasising and planning staff support on a regular basis.

## INTRODUCTION

First‐line managers (FLMs) are responsible for the quality of care their staff members provide to the older people in their care. Home‐ and residential‐care staff are expected to be prepared and have the time to address older people's physical, social, psychological and existential needs. However, research has long shown that it is challenging for staff to find time in their daily work for conversations about existential concerns [1, 2]; moreover, due to increased time constraints, this challenge has increased over time. Aside from limited time, staff members have reported that insecurity, fear and lack of knowledge limit their ability to address these issues [3, 4]. Existential concerns often arise in vulnerable situations when thoughts about meaning and meaninglessness surface and having these thoughts remains unmet or unaddressed can contribute to existential loneliness [5]. FLMs play an important role in leading and organising care provision and supporting and guiding staff. However, there is little knowledge of FLMs' views on staff members' challenges and their need for support when encountering older people's existential concerns.

Becoming old is a complex process and a unique experience for each person. Older people are not a homogeneous group but age differently. Most older people enjoy life and wish for it to continue as long as possible [6], but becoming old can be seen as an ontological challenge, in addition to involving physical and cognitive challenges [7]. Older people's experience of an ageing body that may lead to frailty and unpredictability can evoke feelings of being trapped in an unknown body [8, 9]. Aside from losses and declining health [10], old age entails an increased desire to turn inwards and reflect on how life has turned out [11, 12], and thoughts about meaning in life may arise. Martela and Steger [13] described three dimensions of meaning in life: coherence (i.e. making sense of the world and one's own life); purpose (i.e. having a direction and future‐oriented goals) and significance (i.e. concerning values, worth and importance). The meaning of being old can encompass contradictions and paradoxes, such as embracing both weakness and strength, and regret and reconciliation [12]. Yet being dependent on others—such as needing care and lacking control—can lead to vulnerability [14], while being unrecognised or met with indifference can cause suffering, alienation and existential loneliness [9, 15]. A conceptual review of qualitative studies of loneliness defined ‘existential loneliness’ as a feeling of fundamental separateness from others and the world and as an absence of meaningful relationships [16]. A scoping review of 121 quantitative and qualitative studies on the conceptualization and operationalization of existential loneliness showed that the most‐shared key component was that existential loneliness was inherent in being human, along with an awareness of separateness, the uniqueness of one's subjective experience and one's aloneness in the universe [17]. A study by McKenna‐Plumley et al. [18] presented similar results but also emphasised that existential loneliness involves existential concerns about one's place and purpose in the world; thus, existential loneliness encompasses existential concerns. Tillich [19] described three areas of existential concerns: fear and death, emptiness and meaninglessness and guilt and condemnation. Similarly, Yalom [20] described four existential concerns relating to human life conditions: death, freedom, isolation and meaninglessness. Acknowledging older people and recognising their existential concerns—that is, thoughts about life, approaching death, meaning and meaninglessness—are therefore crucial parts of caring. Previous research has shown that the experience of existential loneliness can be manageable if it is accepted and acknowledged by others [21, 22]. Acknowledging older people's existential concerns can provide opportunities for existential conversations and be used as a means of easing existential loneliness.

The place of care and the care context can either hinder or support staff in recognising older people's thoughts about existential concerns. According to a Swedish multiple‐case study [23], the care context matters regarding the opportunity to address existential issues and loneliness. When comparing home, residential, hospital and palliative care, aspects of time, place and professional roles differ, which in turn affect the staff members' focus. Time constraints and a fast pace were seen as hindrances, as were discomfort in discussing existential issues and insecurity in addressing them. A Swedish study [24] found that nursing assistants providing end‐of‐life care in a nursing home concentrated on bodily care rather than existential needs. In addition, a study on communicative challenges in home care in Sweden found that older people's existential issues, worries and even frailty were often vaguely expressed and therefore difficult for nursing assistants to grasp [25]. Taken together, these findings indicate a need for support to help staff address older people's existential concerns. Since FLMs' role entails meeting the needs of the older people in their unit and supporting employees, their views on staff challenges when encountering older people's existential concerns are crucial.

### Aim

The aim of this study was to describe FLMs' views on the challenges faced by staff when encountering older people's existential concerns in home and residential care.

### Research questions

This study addresses both qualitative and quantitative research questions.

#### Qualitative research questions

What are FLMs' views on (a) older people's existential loneliness, (b) conversations about existential concerns between staff and older people, (c) conversations about existential concerns among colleagues, (d) hindrances to existential conversations and (e) supporting staff and hindrances to supporting staff in dealing with existential concerns?

#### Quantitative research questions

Are there differences in the support provided to staff in addressing existential concerns (a) between home care and residential care, (b) in relation to the FLMs' professional education and (c) depending on the number of employees the FLM oversees?

## METHODS

### Design

This study has a cross‐sectional design involving a combination of qualitative and quantitative methods, based on a randomly selected sample of FLMs in Swedish municipal care and service for older people. An online structured questionnaire that was specifically designed for this study was used to collect information at the national level. This study is part of the LONE study (Existential Loneliness among Frail Older People) [26], which aims to explore existential loneliness from various perspectives. The development of the questionnaire was inspired by earlier results from the LONE study showing that FLMs play an important role in care provision and that care prerequisites differ between contexts [23].

### Context

In Sweden, FLMs hold the overall responsibility for care quality, administration, budgeting, staff management and the work environment, which includes providing support to staff [27]. Each FLM is responsible for an average of 60 direct‐care staff (i.e. nursing assistants). Registered nurses are responsible for nursing and medical care and are not subordinate to the FLMs, as they belong to a different organisational structure.

There is a stated principle in Sweden of helping older people remain in their own homes for as long as possible, which is known as ‘ageing in place’; thus, even very complex care can be provided at home in Sweden [28]. When an older person's needs can no longer be met at home, residential care may be required. In Sweden, care for older people is primarily a tax‐financed municipal responsibility, although it can be executed by private actors or non‐profit organisations.

### Data collection and participants

The inclusion criteria for the study were being an FLM in municipal care and service to older people, and holding responsibility for staff. Simple random sampling [29, 30] was used in which care units were identified from the National Board of Health and Welfare website [31]. Every eighth unit was randomly selected from a register of 4058 home‐ and residential‐care units in Sweden, for a total of 504 selected units. An e‐mail message was sent to the FLMs of the 504 units; 467 managers received the message and were considered eligible participants. The e‐mail included links to an online self‐administered questionnaire, a 2‐minute introductory video and an information letter; the letter explained the research purpose that participation was voluntary and how responses would be handled and stored according to legislation. Data were collected in May and June 2019. Initially, 56 participants completed the questionnaire after the first e‐mail message; three reminders were then sent, leading to an additional 81 responses. In total, 137 participants were included in the study. One participant was excluded for not having responsibility for staff, resulting in a response rate of 29%.

### Questionnaire

We found no existing questionnaire that covered the existential needs of older people or the associated need for staff support from the FLM's perspective; therefore, a questionnaire was developed based on previous empirical findings on existential loneliness (e.g. [23]). The questionnaire was tested during a seminar with experienced research colleagues; after revision, it was tested again, using the think‐aloud method [30], by a person with prior FLM experience. As a final test of the questions and response alternatives, the researchers in the LONE study completed the questionnaire online, and their comments were used to make further revisions. The questionnaire included a total of 41 items about (1) the unit, (2) the FLM's views on existential loneliness and conversations about existential concerns as well as hindrances of such conversations, (3) the FLM's views on support provided to staff in addressing existential concerns, (4) volunteer engagement and (5) demographics about the FLM. The questionnaire included statements to be responded to using an adjectival response scale with continuous responses, as well as questions with fixed‐response alternatives such as ‘Yes’, ‘No’ and ‘I don't know’. Some of the items had multiple‐choice response alternatives to which the managers could add alternatives. (The questionnaire is appended as a supplementary file—Data S1) Due to a technical error, the response alternatives for one item about existential loneliness were reversed, so the quantitative results in relation to this item were not included in the results. There were also six open‐ended questions that allowed the respondents to provide additional information with no word limits. The results regarding volunteers will be presented elsewhere.

### Analysis

The qualitative analysis was based on the texts provided in response to the open‐ended questions—a total of 7280 words; these were analysed using a manifest content analysis [32]. First, the texts were grouped relative to each open‐ended question. Within each group, each statement was labelled by the first and last authors separately. Thereafter, in constant alternation between the labels and the text, the labels were discussed and sorted into categories based on their meaning; the categories were then discussed among all four authors. Quotations from the texts are used to illustrate the presentation of the results.

The quantitative analysis consisted of descriptive statistics (percentages) used to present demographics and frequencies. Pearson's chi‐squared test [33] was used to compare support provided to staff by the following demographics: having responsibility for home care versus residential care; the FLMs' professional backgrounds (social work/registered nurse/other) and the number of employees managed by each FLM (i.e. 1–30, 31–50, ≥51). Statistical analyses were performed using IBM SPSS version 28 (IBM Corp., Armonk, NY, USA).

### Ethics

This study is part of the LONE study, which was approved by the Swedish Ethical Review Authority (ref. number 2014/652); the guidelines of the Helsinki Declaration were followed [34].

## RESULTS

The FLMs (*n* = 136) had a mean age of 51 years. Most had an educational background in social work (41%) or nursing (25%), while 34% reported other educational backgrounds, such as occupational therapy, management, behavioural science and social science. Over half of the managers (67%) had more than 5‐year experience as managers. Of the respondents, 53% were responsible for 31–50 employees and 21% were responsible for over 50 employees (Table 1).

**TABLE 1:** Descriptions of first‐line managers and units, *n* = 136.

Characteristics		
Age, years^a^		
Range, mean (SD)	27–74	51 (9.5)
	***n***	**(%)**
Gender^b^		
Women	111	(83)
Men	21	(16)
Other	1	(1)
Professional education^c^		
Social work	55	(41)
Registered nurse	34	(25)
Occupational therapist	6	(4)
Physiotherapist	1	(1)
Other reported education (e.g. management, behavioural science and social science)	39	(29)
Work experience as a manager in health and social care^b^		
<1 year	7	(5)
1–5 years	37	(28)
6–10 years	30	(23)
>10 years	59	(44)
Work experience as a manager in the current setting		
<1 year	18	(13)
1–5 years	87	(64)
6–10 years	16	(12)
>10 years	15	(11)
Care setting^d^		
Home care	68	(50)
Senior housing	9	(7)
Group dwelling	7	(5)
Residential care	71	(52)
Number of employees		
1–10	1	(1)
11–30	35	(26)
31–50	72	(53)
51–70	16	(12)
>70	12	(9)
Funding		
Public	112	(82)
Private	23	(17)
Non‐profit	1	(1)

^a^ Missing = 8.

^b^ Missing = 3.

^c^ Missing = 1.

^d^ More than one alternative was possible.

### First‐line managers' views on older people's existential loneliness

Of the FLMs, 11% reported that older people in their care units frequently expressed existential loneliness, while 77% reported that they sometimes did so (Table 2). The FLMs described existential concerns and existential loneliness as being about meaning and life, death and dying, loss, freedom, autonomy and missing joy in life. Other descriptions involved missing relations with others and not being understood; for example, having a religious belief that others have difficulty relating to. One FLM cited an example, saying that existential loneliness could arise ‘when living or being in an environment that does not give energy, and where fellow humanity, competence, joy, empathy and the ability to focus on individual needs is missing’ (Manager no. 70, woman, age 52 years).

**TABLE 2:** First‐line managers' views on older persons' existential loneliness and conversations, *n* = 136.

	*n*	(%)
Older persons express existential loneliness		
Frequently	15	(11)
Sometimes	105	(77)
Rarely	12	(9)
Never	–	
Don't know	4	(3)
Staff have conversations about existential concerns with older persons		
Frequently	17	(13)
Sometimes	92	(68)
Rarely	14	(10)
Never	2	(1)
Don't know	11	(8)
Staff have conversations about existential concerns with colleagues^a^		
Frequently	22	(16)
Sometimes	78	(58)
Rarely	30	(22)
Never	3	(2)
Don't know	2	(1)
Staff have opportunities for individual conversations with older persons^a^		
Frequently	84	(62)
Sometimes	43	(32)
Rarely	8	(6)
Never	–	

^a^ Missing = 1.

### First‐line managers' views on conversations about existential concerns between staff and older people

Of the managers, 13% stated that their staff members often had conversations about existential concerns with the older people in their care, and 68% said that their staff sometimes had such conversations (Table 2). The conversations were said to be about *meaning in life*; *losses and longing for meaningful relations*; *d* *eath, fears and uncertainty* and *supporting a meaningful everyday life*.

Older people's experience of *meaning in life* was reportedly a recurring topic of conversation that concerned life and death as well as the older person's life situation. These conversations were about the sorrows and joys experienced in life, the older person's current life situation and the well‐being of relatives. The older person might express feeling like a burden and might also question the life she or he had lived. Feelings of meaninglessness and of no longer being able to control one's life were also articulated. According to one manager, in such existential conversations, some older people might say that ‘they feel content and “finished” with life’ (Manager no. 90, woman, age 54 years).

The conversations about *losses and longing for meaningful relations* covered the loss of close relations, abilities and bodily functions. Longing for one's spouse, relatives and friends could refer to people who were no longer alive or who lived far away. According to the comments, a lack of meaningful relations was related to loneliness and to a feeling of being in an unfamiliar context. One manager wrote about ‘the lack of meaningful relationships and, in old age, not having many friends left in life; death and fear of death; loneliness even if you are surrounded by people’ (Manager no. 60, woman, age 48 years).

The conversations about *death, fears and uncertainty* were about being at the end of life, dying and death. The FLMs noted that some older people expressed not wanting to live and instead wanting to end their life. The fears and uncertainty might also be about the process of dying and what happens after death. According to the comments, however, not everyone feared death: one manager wrote that ‘some older people do not fear death—it will come when the time is right’ (Manager no. 69, man, age unreported).

The conversations about *supporting a meaningful everyday life* covered life in the here and now. The managers noted the importance of supporting and enabling the older person to do things that created meaning, were forward‐looking and included talking about life and joy. One manager described ‘the importance of being able to do what you can do, as long as you want and can, and with support’ (Manager no. 26, woman, age 44 years).

### First‐line managers' views on conversations about existential concerns among staff

Of the FLMs, 16% stated that their staff often had conversations with their colleagues about existential concerns, and 58% said that their staff sometimes had such conversations (Table 2). The FLMs' views on conversations about existential concerns among staff members involved *how to encounter existential concerns*; *reflections on the older person's situation*; *how to promote meaningfulness based on shared core values* and *how caring was affected by and affected their own experiences and feelings*.

The conversations among staff about *how to encounter existential concerns* primarily concerned difficulties and having the courage to encounter the older people and their relatives, especially towards the end of life. This might be the case when the older person expressed that life should be over or questioned whether life was worth living. One manager mentioned ‘the difficulty of talking about what is most important, and lack of experience [doing so]’ (Manager no. 91, woman, age 59 years).

The staff's *reflections on the older person's situation* included becoming old and experiencing dementia, loneliness and death. Some older people said that they felt satisfied, while others expressed distress and needed to talk about life and death—a difference that the staff reflected on. The conversations among colleagues included reflections about what it would be like to be old and continually require support from others. One manager commented that loneliness might not be a problem for the older person but that sometimes staff members might think that it was from their own perspective. Another manager wrote that ‘[the staff] mostly reflect on the importance of being aware and open to the need for existential conversations’ (Manager no. 37, man, age 55 years).

The conversations among staff members about *how to promote meaningfulness based on shared core values* concerned both the older person and her or his family and were about how to create a sense of meaningfulness and a feeling of being at home, and how to promote and preserve a healthy and independent life. The conversations also concerned how to find value and meaning in life, such as in one's life story. One manager wrote: ‘We need to talk about existential issues to be able to work in a person‐centred way’ (Manager no. 83, woman, age 56 years).

The conversations about *how caring was affected by and affected their own experiences and feelings* involved the staff members' own experiences of being ill themselves or caring for a relative. In such conversations, staff members shared their life experiences and used them as a basis for understanding the older person's situation. Among staff, caring for someone at the end of life often prompted feelings, thoughts, reflections and conversations about what it was like to encounter the older person's existential concerns and fears of dying. One manager wrote: ‘When an older person has passed away, there are thoughts about death, thoughts about you as a staff member coming home to someone who is dead. How will I as a staff member react then?’ (Manager no. 67, woman, age 45 years).

### First‐line managers' views on hindrances to existential conversations

Regarding major hindrances that prevented older people from having existential conversations, 85% of the FLMs identified impaired cognition (e.g. dementia and stroke) and 51% identified aphasia as problems. Of the managers, 66% reported that unwillingness on the part of the older people impeded talk about existential concerns, and 59% reported insecurity as a hindrance.

**FIGURE 1:**
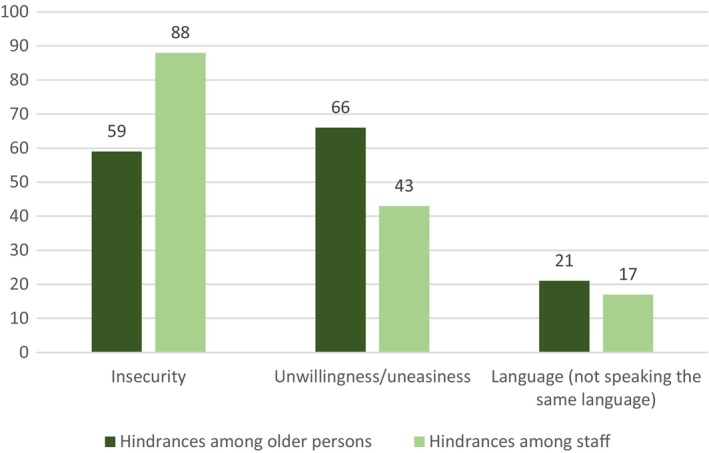
First‐line managers' views on hindrances to having conversations about existential concerns on the part of older persons and staff (%).

Regarding major hindrances that prevented staff from having existential conversations, insecurity was identified by 88% of managers, followed by unwillingness or uneasiness, which was identified by 43% (Figure 1). Of the managers, 57% stated that they had staff with limited ability to speak Swedish, and some estimated that up to 40% of their staff had language limitations. One manager highlighted how difficult it was when not everyone understands and shares the view that older people have existential needs, which was described as being ‘related to cultural and language barriers’ (Manager no. 109, man, age 48 years).

### First‐line managers' views on providing support and on hindrances to support

Most of the FLMs (73%) stated that their staff received support in addressing existential concerns among the older people they cared for. Of the managers, 65% stated that this involved individual support from the manager, while 52% said that this concerned individual support from registered nurses. Just 6% reported that clinical supervision was regularly provided at the unit. There were no differences in the support provided in home care versus that provided in residential care (*p =* 0.538), or in relation to the FLM's professional education (*p* = 0.482) or the number of employees overseen by the FLM (*p* = 0.467) (Table 3).

**TABLE 3:** First‐line managers' views on support for staff, *n* = 136.

	*n*	(%)
Support in encountering existential concerns^a^		
Yes	98	(73)
No	36	(27)
Form of support provided^b^		
Clinical supervision on a regular basis	8	(6)
Education	37	(27)
Structured reflection	49	(36)
Support from the manager	88	(65)
Support from registered nurses	71	(52)

Support provided to staff. subgroup analysis				
	Yes/No	Total	*p*‐value^c^	
Type of care facility				
Home care	41/18	59		0.538
Residential care	44/15	59		
		118^d^		
Professional education				
Social work	39/15	54		0.482
Registered nurse	27/6	33		
Other^e^	23/10	33		
		120^d^		
Number of employees				
1–30	26/10	36		0.467
31–50	49/21	70		
>51	23/5	28		
		134^a^		

^a^ Missing = 2.

^b^ More than one alternative was possible.

^c^ Tested with Pearson's chi‐squared test.

^d^ Missing = 16–18.

^e^ Other educational backgrounds, such as occupational therapy, management, behavioural science and social science.

In the comments, one manager gave examples of the support provided: ‘A mixed bag—we offer them supervision if needed and individual conversations if they so desire, though not regularly’ (Manager no. 1, man, age 38 years). The FLMs also described other forms of support, such as supervision on special occasions when needed and the opportunity to contact a counsellor or psychologist at the local healthcare centre, the occupational healthcare service or the Swedish Church.

The FLMs were asked to describe what kind of additional support they thought their staff needed. Their answers mainly referred to opportunities for reflection and a supportive and open climate in the work group, which would help the staff members to be confident and flexible in their daily work. The managers described a need for knowledge of the ageing process and skills in communication methodology; one manager wrote: ‘Therapeutic communication techniques—the courage to listen. Adequate knowledge to be able to be professional’ (Manager no. 18, woman, age 66 years).

The FLMs were further asked to describe organisational hindrances to providing support. They mainly mentioned limited financial resources, limited time and logistic difficulties, emphasising their own limited opportunities due to the design and scope of their management assignment, collaboration and organisational structures. One manager commented: ‘Shortage of resources … The message from management is that finances take precedence over quality’ (Manager no. 72, woman, age 59 years). A lack of specially trained clinical supervisors who could address existential concerns was reportedly another hindrance. However, other FLMs claimed that there were no hindrances but that it was instead a matter of prioritising. One manager wrote: ‘We are a small organization, so there are no hindrances. We might broach the subject in a general way at a staff meeting and in the context of supervision’ (Manager no. 108, woman, age 72 years).

## DISCUSSION

The main findings of our study show that the great majority of FLMs reported that the older people in their care units expressed existential loneliness and that staff had conversations about existential concerns with these older people. Most managers also reported that their staff members discussed existential concerns with one another and that they received support in this, albeit mainly on an individual basis, which is a surprisingly positive result. However, the FLMs reported hindrances to existential conversations that affected the staff and the older people in their care.

The FLMs were aware that the older people in their units experience and express existential loneliness, which required the staff to be ready and competent to encounter related questions. The results show that existential conversations between staff and older people were reported by the FLMs to involve topics such as meaning in life; losses and longing for meaningful relations and death, fear and uncertainty. However, previous research has shown that these aspects can be difficult for staff to encounter. Even though staff members experience conversations about existential concerns as meaningful, they simultaneously express fear and difficulty in encountering existential issues [3]. In a European multicentre study including staff from five countries, the staff members expressed a need to improve their sensitivity regarding existential conversations and their self‐confidence to engage in them, and to improve their knowledge and understanding of ageing, death and dying [4]. This finding is in line with the present results, which show that the FLMs perceived the most common hindrance to existential conversations on the part of staff to be insecurity. Since concerns about existential issues are common, and since existential conversations are difficult to carry out, FLMs need to equip their staff with the competence required to cope with this task. Regularly having existential conversations within the staff group could be a first step towards reducing such insecurity and promoting a work climate that allows existential aspects of care to be addressed. Notably, over half of the FLMs stated that limited ability to speak Swedish was common among their staff, complicating the design of possible interventions. As limited ability to speak Swedish is an aggravating factor, constantly striving to improve the conversational skills of staff is essential, not only for practical daily matters but also to address older people's existential concerns.

In addition to staff insecurity as a hindrance to existential conversations, the difficulties older people in residential and home care might have in verbally expressing their thoughts and emotions were described as hindering such conversations. As shown in the results, the FLMs identified impaired cognition and aphasia as major hindrances that in turn affected the possibility of staff identifying existential concerns among the older people in their care. This result is in line with the work of Sundström et al. [3], in which professionals said that some older people were unable—or did not know how—to express their existential loneliness in words. Carr and Fang [35] noted the importance of having an emotional language with which to express existential loneliness, highlighting the importance of care professionals being aware of subtle signs and of discerning and interpreting unspoken messages. In the present results, the FLMs also perceived that older people's unwillingness and uneasiness could prevent them from having conversations about existential concerns. This finding can be contrasted to an inside view from the perspective of older people in nursing homes, which showed that older people expressed feeling existentially trapped in themselves and their bodies and often felt locked away from the outside world [36]. Nyström [37] concluded that staff working with patients suffering from aphasia should spare no effort to understand the patients' existential situation. This perspective shows the importance of staff having the ability to listen and reflect, to perceive the older person's perspective and life world and to take the existential aspects of care into consideration. The FLM's role in enabling an open work climate and ensuring that staff have adequate competence and skills is important.

FLMs play a critical role in emphasising and planning staff support, which can counteract staff insecurity and encourage existential conversations in home and residential care. Although staff members need support when encountering existential concerns among older people, there seemed to be a lack of structured support, as the staff only occasionally received such support, which mainly occurred in the form of individual support. The FLMs reported that other kinds of support were needed, but stated that a lack of time, money and logistical resources prevented them from providing such support. For example, regularly scheduled structured reflection and clinical supervision were rare, although previous studies (e.g. [38]) have shown that clinical supervision can enhance the ability to build caring relationships and can both support staff and improve care.

One of the hindrances mentioned by the managers was a lack of specially trained clinical supervisors who could address existential concerns. Moreover, support was mostly provided by the FLMs themselves, even though many managers were individually responsible for many employees. The FLMs mentioned that a supportive and open work climate could help staff members to be confident and flexible in their daily work. According to a study of managers' experiences of ethical problems in municipal elderly care [39], access to adequate resources was key in providing care that complied with laws and policies. The researchers found that financial control systems were insensitive to the needs of older people. A study of leadership [40] found that units assessed as ‘highly person centred’ had leaders who were committed to staff competence, professional development, team support and care quality. Leaders who understand the needs of both older people and staff and who prioritise adequate support and adequate resources are fundamental to providing person‐centred care to older people.

### Methodological considerations

It is well known that digital surveys typically have low response rates. In an attempt to increase the response rate, an introductory video was attached to the first e‐mail, and three reminders were sent. Other alternatives for distributing the survey might have increased the response rate [30], but it is important to consider the strained work situation of the managers. Although the low response rate threatened the external validity of this study, probability sampling was used, and the units were randomly selected to allow the results to be generalizable [30]. The distribution of the managers between home care and residential care corresponded to the national distribution of care units, and the same applies to the managers in this study and to the division between public and private care providers in Sweden. Based on a national survey from 2021, the gender distribution and professional background of the FLMs participating in this study also corresponded to national figures [27], meaning that the sample can be deemed representative. Even with the low response rate, we argue that this study still holds exploratory value. We also stress the value of the combination of qualitative and quantitative methods, which involved using a manifest content analysis of the open‐ended questions as well as descriptive statistics. To increase the trustworthiness of this research, quotations were used [41]. Although the open‐ended answers deepened our understanding of the quantitative data, more studies from a managerial perspective are needed to improve knowledge of the manager's role and the prerequisites for planning care, including the existential aspects of care.

## CONCLUSIONS AND IMPLICATIONS

Older people receiving home and residential care express existential loneliness. To reduce staff insecurity when encountering such expression, regular conversations within the staff group and constant efforts to improve the staff's conversational skills are needed. An additional challenge is that the older people in the staff's care may have difficulty expressing their thoughts and emotions verbally due to illness such as dementia or stroke. Therefore, staff also need the ability to listen to and reflect on unspoken communication, in order to perceive older people's perspective and life world. To counteract staff insecurity and encourage existential conversations in home and residential care, it is essential for FLMs to understand the needs of both the older people in their care units and the staff they are responsible for, and to recognise their own role in emphasising and planning regular staff support and promoting a work climate that allows existential aspects of care to be addressed.

## AUTHOR CONTRIBUTIONS

M.S., K.B., M.R. and A.K.E. all contributed to designing the study. M.S. had the main responsibility for developing and distributing the questionnaire and for analysing the data together with A.K.E. M.R. and K.B. provided critical input in this process. M.S. wrote the initial draft of the manuscript, and K.B., M.R. and A.K.E. commented critically on and contributed substantially to the manuscript. All authors read and approved the final version of the manuscript.

## FUNDING INFORMATION

This study was funded by the Research Platform for Collaboration for Health, Kristianstad University, and the Vårdal Foundation (Grant 2014‐0127), Sweden.

## CONFLICT OF INTEREST STATEMENT

The authors have no conflict of interest to declare.

## ETHICS STATEMENT

The study was approved by the Swedish Ethical Review Authority (ref. number 2014/652) and followed the guidelines of the Helsinki Declaration.

## Supporting information


Data S1.

## Data Availability

Data have limited availability as they are in Swedish but can be made available on request. Please contact the first author for information.

## References

[1] Beck I , Törnquist A , Broström L , Edberg AK . Having to focus on doing rather than being: nurse assistants' experience of palliative care in municipal residential care settings. Int J Nurs Stud. 2012;49(4):455–464. 22079261 10.1016/j.ijnurstu.2011.10.016

[2] Norell Pejner M , Ziegert K , Kihlgren A . Trying to cope with everyday life: emotional support in municipal elderly care setting. Int J Qual Stud Health Well‐Being. 2012;7(1):19613–19617. 10.3402/qhw.v7i0.19613 PMC3522874 23237630

[3] Sundström M , Edberg AK , Rämgård M , Blomqvist K . Encountering existential loneliness among older people: perspectives of health care professionals. Int J Qual Stud Health Well‐Being. 2018;13(1):1474673. 29869590 10.1080/17482631.2018.1474673 PMC5990949

[4] Edberg AK , Trogu G , Manattini A , Renn‐Żurek A , Modrzejewska DM , Woźnicka EB , et al. Existential loneliness among older people from the perspective of health care professionals: a European multicenter study. Psychol Res Behav Manag. 2023;20(16):2241–2252. 10.2147/PRBM.S408547 PMC10290471 37359147

[5] Sand L , Strang P . Existential loneliness in a palliative home care setting. J Palliat Med. 2006;9(6):1376–1387. 17187546 10.1089/jpm.2006.9.1376

[6] Fjell A , Eriksen K , Hermann M , Boström A , Cronfalk S . Older people living at home: experiences of healthy ageing. Prim Health Care Res Dev. 2021;22:E6. 33658085 10.1017/S1463423621000049 PMC8060837

[7] Shaw RL , West K , Hagger B , Holland CA . Living well to the end: a phenomenological analysis of life in extra care housing. Int J Qual Stud Health Well‐Being. 2016;11(1):31100. 27172516 10.3402/qhw.v11.31100 PMC4864845

[8] Ebrahimi Z , Wilhelmson K , Eklund K , Moore CD , Jakobsson A . Health despite frailty: exploring influences on frail older adults' experiences of health. Geriatr Nurs. 2013;34(4):289–294. 23669314 10.1016/j.gerinurse.2013.04.008

[9] Sjöberg M , Beck I , Rasmussen BH , Edberg AK . Being disconnected from life: meanings of existential loneliness as narrated by frail older people. Aging Ment Health. 2018;22(10):1357–1364. 28714734 10.1080/13607863.2017.1348481

[10] Baltes PB , Smith J . New frontiers in the future of aging: from successful aging of the young old to the dilemmas of the fourth age. Gerontology. 2003;49(2):123–135. 12574672 10.1159/000067946

[11] Andersson M , Hallberg IR , Edberg AK . Old people receiving municipal care, their experiences of what constitutes a good life in the last phase of life: a qualitative study. Int J Nurs Stud. 2008;45(6):818–828. 17540379 10.1016/j.ijnurstu.2007.04.003

[12] Santamäki Fischer R , Norberg A , Lundman B . Embracing opposites: meanings of growing old as narrated by people aged 85. Int J Aging Hum Dev. 2008;67(3):259–271. 19049246 10.2190/AG.67.3.d

[13] Martela F , Steger M . The three meanings of meaning in life: distinguishing coherence, purpose, and significance. J Posit Psychol. 2016;11(5):531–545.

[14] Abley C , Bond J , Robinson L . Improving interprofessional practice for vulnerable older people: gaining a better understanding of vulnerability. J Interprof Care. 2011;25(5):359–365. 21740093 10.3109/13561820.2011.579195

[15] Svanström R , Johansson Sundler A , Berglund M , Westin L . Suffering caused by care: elderly patients' experiences in community care. Int J Qual Stud Health Well‐Being. 2013;8(1):20603. 24262375 10.3402/qhw.v8i0.20603 PMC3837301

[16] Mansfield L , Victor C , Meads C , Daykin N , Tomlinson A , Lane J , et al. A conceptual review of loneliness in adults: qualitative evidence synthesis. Int J Environ Res Public Health. 2021;18(21):11522. 34770035 10.3390/ijerph182111522 PMC8582800

[17] Gil Álvarez M , Haugan G , Larsson H , Saarelainen S‐M , Duppen D , Dezutter J . Mapping existential loneliness: a scoping review on existential loneliness/isolation conceptualizations and operationalizations. J Humanist Psychol. 2023:1–25.

[18] McKenna‐Plumley P , Turner R , Yang K , Groarke J . ‘It's a feeling of complete disconnection’: experiences of existential loneliness from youth to older adulthood. BMC Psychol. 2023;11(1):408. 37990348 10.1186/s40359-023-01452-4 PMC10664587

[19] Tillich P . The courage to Be. New Haven: Yale University Press; 1952.

[20] Yalom I . Existential psychotherapy. New York: Basic Books; 1980.

[21] Wijesiri M , Samarasinghe K , Edberg AK . Loneliness among older people living in care homes in Sri Lanka. Int J Older People Nursing. 2019;14(4):e12253. 10.1111/opn.12253 31274242

[22] Sjöberg M , Edberg AK , Rasmussen BH , Beck I . Being acknowledged by others and bracketing negative thoughts and feelings: frail older people's narrations of how existential loneliness is eased. Int J Older People Nursing. 2019;14(1):e12213. 10.1111/opn.12213 30403002

[23] Sundström M , Blomqvist K , Edberg AK , Rämgård M . The context of carematters: Older people’s existential loneliness from the perspective of healthcare professionals. A multiple case study. Int J Older People Nurs. 2019;14(3):e12234. 31025806 10.1111/opn.12234

[24] Holmberg B , Hellström I , Österlind J . End‐of‐life care in a nursing home: assistant nurses' perspectives. Nurs Ethics. 2019;26(6):1721–1733. 29950147 10.1177/0969733018779199

[25] Sundler AJ , Eide H , van Dulmen S , Holmström IK . Communicative challenges in the home care of older people: a qualitative exploration. J Adv Nurs. 2016;72(10):2435–2444. 27144778 10.1111/jan.12996

[26] Edberg AK , Bolmsjö I . Exploring existential loneliness among frail older people as a basis for an intervention: protocol for the development phase of the LONE Study. JMIR Res Protoc. 2019;8(8):e13607. 31414663 10.2196/13607 PMC6712957

[27] National Board of Health and Welfare . Prerequisites and Support for First‐Line Managers 2021. [In Swedish: Socialstyrelsen. Förutsättningar och stöd för första linjens chefer 2021. Kartläggning av första linjens chefer i äldreomsorgen]. Stockholm: National Board of Health and Welfare; 2021.

[28] National Board of Health and Welfare . Care of Older People: Progress report 2019 (annual report) [in Swedish: Vård och omsorg om äldre—Lägesrapport 2019]. Stockholm: National Board of Health and Welfare; 2019 https://www.socialstyrelsen.se

[29] Dillman DA , Smyth JD , Christian LM . Internet, phone, mail, and mixed‐mode surveys: the tailored design method. 4th ed. Hoboken, NJ: Wiley; 2014.

[30] Polit DF , Beck CT . Nursing research: generating and assessing evidence for nursing practice. 9th ed. Philadelphia, PA: Wolters Kluwer/Lippincott; 2012.

[31] National Board of Health and Welfare . National survey of municipal health and social Care of Older Persons 2018. Swedish: Öppna jämförelser 2018—Vård och omsorg om äldre. Stockholm: National Board of Health and Welfare; 2018 https://www.socialstyrelsen.se

[32] Graneheim UH , Lindgren BM , Lundman B . Methodological challenges in qualitative content analysis: a discussion paper. Nurse Educ Today. 2017;56:29–34. 28651100 10.1016/j.nedt.2017.06.002

[33] Norman GR , Streiner DL . Biostatistics: the bare essentials. Shelton: PMPH‐USA Ltd.; 2014.

[34] World Medical Association . Declaration of Helsinki: ethical principles for medical research involving human subjects. Ferney‐Voltaire: World Medical Association; 2013. https://www.wma.net/policies‐post/wma‐declaration‐of‐helsinki‐ethical‐principles‐for‐medical‐research‐involving‐human‐subjects/

[35] Carr S , Fang C . A gradual separation from the world: a qualitative exploration of existential loneliness in old age. Ageing Soc. 2021;43(6):1436–1456.

[36] Österlind J , Ternestedt BM , Hansebo G , Hellström I . Feeling lonely in an unfamiliar place: older people's experiences of life close to death in a nursing home. Int J Older People Nursing. 2017;12(1):e12129. 10.1111/opn.12129 27624362

[37] Nyström M . Professional aphasia care: trusting the patient's competence while facing existential issues. J Clin Nurs. 2009;18(17):2503–2510. 19619206 10.1111/j.1365-2702.2009.02825.x

[38] Bergdahl E , Benzein E , Ternestedt BM , Andershed B . Development of nurses' abilities to reflect on how to create good caring relationships with patients in palliative care: an action research approach. Nurs Inq. 2011;18(2):111–122. 21564392 10.1111/j.1440-1800.2011.00527.x

[39] Jonasson LL , Sandman L , Bremer A . Managers' experiences of ethical problems in municipal elderly care: a qualitative study of written reflections as part of leadership training. J Healthc Leadersh. 2019;11:63–74. 31213938 10.2147/JHL.S199167 PMC6549386

[40] Backman A , Sandman PO , Sköldunger A . Characteristics of nursing home units with high versus low levels of person‐centred care in relation to leadership, staff‐ resident‐ and facility factors: findings from SWENIS, a cross‐sectional study in Sweden. BMC Geriatr. 2021;21:498. 34530734 10.1186/s12877-021-02434-0 PMC8447583

[41] Guba EG . Criteria for assessing the trustworthiness of naturalistic inquiry. Educ Commun Technol. 1981;29(2):75–91.

