# Supplementary material for: Challenges for staff encountering older people's existential concerns: Swedish first‐line managers' views. A cross‐sectional study
Source: Scand J Caring Sci. 2024 Nov 20;39(1):e13300. doi: 10.1111/scs.13300 (PMC11686042; doi:10.1111/scs.13300)
Supplement: Supplementary file 1 — Data S1. [file SCS-39-0-s001.docx]

1. QUESTIONS ABOUT THE UNIT

1.1 **In what type of care facility do you work as a first-line manager?**^1^

Home care

Serviced apartments

Group living

Residential care facilities

Sheltered housing

Other: ……………………………………………………………………………………………………………………….

1.2 **Does your unit have any specialization**?^1^

Dementia care

Short-term care

Respite care

Palliative care

Other: ……………………………………………………………………………………………………………………….

1.3 **What type of organization runs the unit in which you work as a first-line manager?**

Public provider

Private provider

Cooperative, foundation, NGO or similar provider

1.4 **How many people do you have personnel responsibility for?**

1–10

11–30

31–50

51–70

More than 70

Have no personnel responsibility

1.5 **What categories of staff are you responsible for**?**^1^**

Nursing assistants

Licensed practical nurses

Registered nurses

Occupational therapists

Physiotherapists

Maintenance staff

1.6 **Are there employees in your unit who have limited ability to speak Swedish?**

Yes

No

Don’t know

If yes, state the approximate share in per cent ………………………………………………………….

^1^ More than one alternative is possible.

1.7 **Are there older people in your unit who speak a language that no one on the staff is fluent in?**

Yes

No

Don’t know

2. QUESTIONS ABOUT EXISTENTIAL LONELINESS AND EXISTENTIAL CONCERNS

**You associate existential loneliness (a deeper kind of loneliness) with:**

2.1 Life, meaning^2^

2.2 Dying, death, loss

2.3 Freedom, autonomy, self-determination

2.4 Loneliness, lack of relationships

2.5 Other: …………………………………………………………………………………………………………………….

2.6 **Are there older people in your unit who express existential loneliness?**

Often

Sometimes

Seldom

Never

Don’t know

2.7 **Do the staff members have conversations about existential concerns with the older people?**

Often

Sometimes

Seldom

Never

Don’t know

2.8 **If the staff members have conversations about existential concerns with the** **older people, please give examples of what these conversations are about?**

……………………………………………………………………………………………………………………………………….

2.9 **Do the staff members have conversations about existential concerns?**

Often

Sometimes

Seldom

Never

Don’t know

2.10 **If the staff members have conversations among themselves about existential concerns,**

**please** **give examples of what the conversations are about?**

…………………………………………………………………………………………………………………………………….

^2^ Has not been included in the results since the response alternatives were incorrectly reversed.

2.11 **If there are hindrances preventing *older people* from having existential conversations, what are these hindrances?**^1^

Reduced cognitive function (e.g. dementia diseases or stroke)

Aphasia

Insecurity about existential conversations

Unwillingness to talk about existential concerns

Language (not speaking the same language)

Other: ………………………………………………………………………………………………………………………….

2.12 **If there are hindrances preventing *staff* from having existential conversations, what are these hindrances?**^1^

Insecurity about existential conversations

Unwillingness to talk about existential concerns

Language (not speaking the same language)

Other: ……………………………………………………………………………………………………………………………

3. QUESTIONS ABOUT THE STAFF

3.1 **Do the staff have opportunities for everyday individual conversations with the older people?**

Often

Sometimes

Seldom

Never

3.2 **Are staff given support in encountering and having conversations about existential concerns with older people?**

Yes

No

3.3 **If yes, how is it provided?**^1^

Supervision on a regular basis

Structured reflection

Individual conversations with first-line manager

Individual conversations with registered nurse

Education/training

Other: …………………………………………………………………………………………………………………………….

3.4 **What additional support do you think the staff would need?**

………………………………………………………………………………………………………………………………………..

3.5 **Are there hindrances in the organization to giving such support? If so, what are they?**

…………………………………………………………………………………………………………………………………………

^1^ More than one alternative is possible.

4. QUESTIONS ABOUT VOLUNTEERS (13 questions, will be presented elsewhere)

5. QUESTIONS ABOUT YOU AS FIRST-LINE MANAGER

5.1 **What is your position?**

First-line manager

Area manager

Head of social services

Other………………………………………………………………………………………………………………………………

5.2 **Are you:**

Female

Male

Other

5.3 **What is your age?** ........................................................................................................

5.4 **Do you have a professional education with an academic degree?**

Yes

No

If yes, which professional education?

Social worker/social service

Registered nurse

Occupational therapist

Physiotherapist

Other; please specify …………………………………………………

5.5 **Do you have leadership education/training?**

Yes

No

5.6 **How much experience do you have as a manager/leader in health and social care?**

Less than 1 year

1–5 years

6–10 years

More than 10 years

5.7 **How much experience do you have as a first-line manager at this unit?**

Less than 1 year

1–5 years

6–10 years

More than 10 years

6. OTHER QUESTIONS

6.1 **Other comments or reflections**

………………………………………………………………………………………………………………….
